# Wear Resistance of the Glass-Fiber Reinforced Polymer Composite with the Addition of Quartz Filler

**DOI:** 10.3390/ma14143825

**Published:** 2021-07-08

**Authors:** Wojciech Zurowski, Jarosław Zepchło, Aneta Krzyzak, Edwin Gevorkyan, Mirosław Rucki, Elżbieta Siek, Anita Białkowska

**Affiliations:** 1 Faculty of Mechanical Engineering, Kazimierz Pulaski University of Technology and Humanities in Radom, ul. Stasieckiego 54, 26-600 Radom, Poland; wojciech.zurowski@uthrad.pl; 2 RADWAG, ul. Toruńska 5, 26-600 Radom, Poland; zepchlo.jaroslaw@gmail.com; 3 Faculty of Aeronautics, Military University of Aviation, 08-521 Dęblin, Poland; a.krzyzak@law.mil.pl; 4 Department of Quality, Standardization, Certification and Manufacturing Technology, Ukraine State University of Railway Transport, 7 Feuerbach sq., 61010 Kharkiv, Ukraine; cermet-u@mail.com; 5 Faculty of Economics and Finance, Kazimierz Pulaski University of Technology and Humanities in Radom, ul. Chrobrego 31, 26-600 Radom, Poland; e.siek@uthrad.pl; 6 Faculty of Chemical Engineering, Kazimierz Pulaski University of Technology and Humanities in Radom, ul. Chrobrego 27, 26-600 Radom, Poland; a.bialkowska@uthrad.pl

**Keywords:** polymer composite, resin, glass fiber reinforcement, silica filler, quartz powder, wear, tribotester

## Abstract

The paper presents the results of investigations on the glass fiber reinforced composite for the floor panels with quartz powder additions of different percentages in terms of wear resistance, friction coefficient, hardness, and strength. The wear resistance was assessed using the specific wear work parameter determined by the novel tribotester with friction band. It was found that an increase in quartz powder addition to the tested polymer composite does not enhance its mechanical increasingly properties. From the wear tests it can be concluded that only the composite with four layers of glass fibers and 6 wt.% of the quartz powder exhibited improvement of the wear resistance, but its shear strength was lower than that of the two layer specimens with similar powder proportions. On the other hand, the highest friction coefficient’s, which is microhardness *HV_05_*, shear strength and impact strength were attained for the composite with two layers of glass fibers and 3 wt.% of the quartz powder. Among four layer samples, very close results were obtained for the samples with 10% of powder and insignificantly lower strength were observed for the samples with no powder added. The results revealed that there is no clear trend for the effect of silica filler percentage on the composite performance, which indicates the need for individual purpose-dependent decision making in the design of the glass fiber reinforced composites with quartz powder filler.

## 1. Introduction

Mechanical properties of epoxy resin can be considered superior with respect to other thermosets and enables its dominance in composite fabrication as a matrix [1]. Polymer hybrid materials contain fillers or modifiers that have different functionalities, thanks to which they are characterized by unique usable, technological, and processing properties [2]. Epoxies can achieve favorable mechanical properties dependent on the hardeners, but they may additionally be reinforced with glass or carbon fibers [3]. Furthermore, improvement can be made by producing a hybrid epoxy composites with several types of powder or consisting of both powder and fibrous modifiers [4].

From the materials science perspective, the main objective is to develop materials with an improved wear resistance by testing different combinations of reinforcements and fillers in a particular polymer matrix [5]. In critical applications such as aerospace, automotive, marine, and construction the use of epoxy resins with nanofillers is rapidly growing [6]. However, in other cases such as floor panels, it would unnecessarily increase the cost, which motivates the investigations on cheaper materials that allow the improvement of the mechanical properties of a reinforced polymer composite. For example, Kumar et al. utilized as reinforcement the industrial solid waste of ferrochrome slag containing oxides such as Al_2_O_3_, SiO_2_, and MgO [7], while Heriyanto et al. added waste glass powder to the resin-based composites [8]. Even though numerous research indicated the reinforcing capacity of industrial wastes and industrial waste-filled polymer composites being investigated, the large-scale commercial applications have not been reported so far [9].

It is estimated that the glass fibers cover ca. 90% of the market of reinforced plastic composites [10]. As a filler, silica (SiO_2_) is often used either in its pure state or in various combinations with metal oxides to improve various properties of the composite among which are the mechanical properties, as well as abrasion resistance [11]. Among silicon dioxide-based commercially available fillers for polymer composites, the following can be distinguished: glass beads produced by the melting and molding of fine glass powder to obtain spherical particles; foundry sand with spherical particles fabricated out of bauxite by melting in electric-arc furnace and subsequent spraying and cooling; crystalline silica prepared using chemical purification and mechanical fragmentation of the natural quartz; and amorphous fused silica produced by melting crystalline silica, its re-solidification, and subsequent crushing and pulverizing [12]. The term ‘quartz’ is often used instead of ‘crystalline silica’ [13]. In some applications, it is useful to apply silica fillers of two types, e.g., nanosilica beads and microscale ball-milled glass microscope coverslips [14].

However, researchers do not focus on the effect of silica proportion on the wear resistance of a glass fiber reinforced composites. For instance, Kiran et al. investigated mechanical properties of polymer matrix composites with small proportions of fillers including silicon carbide, titanium dioxide, alumina, and titanium carbide added to the composite along with glass fibers [15]. The present study is dedicated to the glass fiber reinforced composite for the floor panels with quartz powder used as filler. Test campaign includes wear tests, determination of coefficient of friction, and hardness measurement.

## 2. Materials and Methods

The tested material was based on a resin MC-DUR 1200VK reinforced with two or four layers of E-type glass mats of a mass per unit area 300 g/m^2^ where silane pre-treatment was applied to the fibers in the form of a powder binder. According to the available data, the Young’s modulus of E-glass is 76 GPa and the tensile failure stress is 3.45 GPa [16]. EM1004 glass mats were delivered by Krosglass S.A. (Krosno, Poland). The mats were made of glass fibers of type E glass with a diameter of 12 µm and a linear weight of 30 tex. Fibers approximately 50 mm long were randomly arranged with respect to each other.

**Figure 1:**
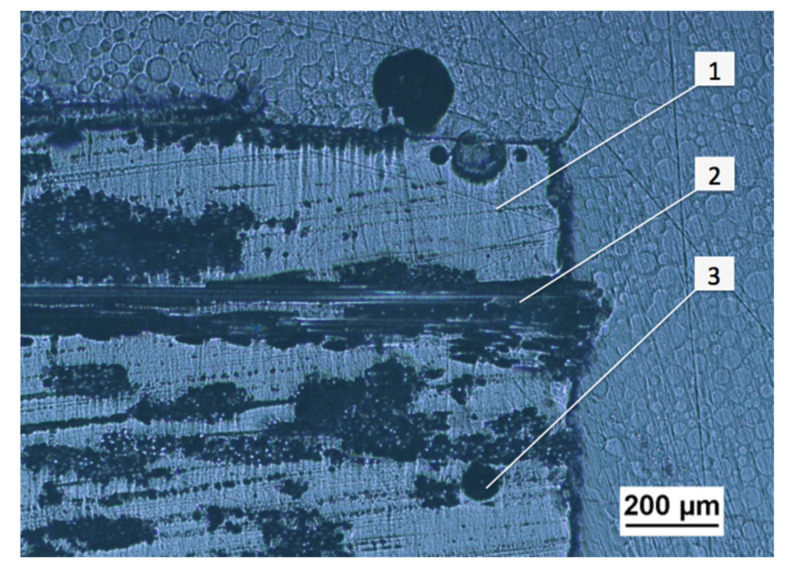
Cross-section of the glass fiber reinforced composite with no quartz powder addition.

The cross-sectional structure of the composite is shown in Figure 1, where the glass fibers denoted (2) are observed in the polymer matrix (1), where some discontinuities may appear in the form of bubbles (3).

Laminates were made by the hand lay-up method. The resin was mixed with the dedicated hardener in a weight ratio 3:1. After lamination, the manufactured plates underwent pressing with a pressure of 2MPa and left for 24 h at room temperature 20 °C for hardening. The experiments were conducted no earlier than after 2 weeks. 

The quartz filler was added in the form of powder and of particle dimensions between 0.1 and 0.3 mm. Its chemical composition was 99.57% SiO_2_, 0.12% Al_2_O_3_, and 0.31% of other oxides. The samples were made with following proportions of the quartz powder: 0%, 1%, 3%, 6%, and 10% by mass. For each proportion of the added powder, 6 samples were made for further tribological tests. The height of the specimens was 30 mm and the dimensions of the area subjected to the friction test were 15 mm width × 1.5 or 2.8 mm thickness depending on the respective number of layers, 2 or 4. 

Wear resistance refers to the working life of the materials during service and it is dependent on the ingredients and manufacturing technique [17]. In tribological assessments of the plastics and elastomers, the wear resistance can be derived from an experimentally determined wear factor dependent on the amount of material removed by wear during a given time period related to the values of load and surface velocity [18]. Since the time, pressure, velocity, and depth units can be different, the wear factor *K* is a relative measure enabling comparison of different materials provided that the operating conditions are the same.

**Figure 2:**
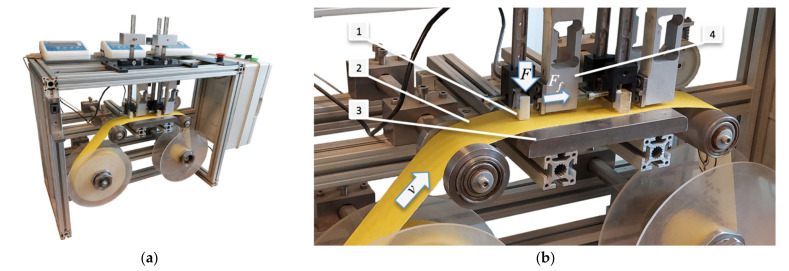
TT-4 tribotester: (**a**) overall view; (**b**) working space.

The tribological tests were performed using a novel device TT-4 shown in Figure 2. It resulted in the removal of the worn material of sliding elements and the avoidance of the worn surfaces participation in the test, since the sample (1) is sliding against the friction belt (2) that moves with certain velocity *v* between the pin and the table (3). The load *F* causes appearance of the friction force *F_f_* registered by the extensometer (4). The measurement data are displayed in real time and simultaneously transferred to the PC using the RS port. 

**Figure 3:**
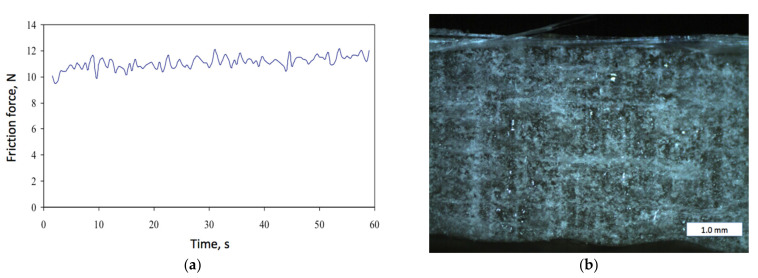
Examples of the friction tests: (**a**) Friction force registered by the TT-4 tester during 60 s of the test; (**b**) the specimen’s surface after friction test: 4-layer composite with 10% of quartz powder.

Figure 3a presents an example of registered friction force *F_f_* for the sample made up of four layers of E-type glass mat with 6 wt.% of quartz powder specified above. In this example, the mean friction force was 11.089 N, while its Root Mean Square value was 11.099 N. In the Figure 3b, a specimen’s surface after the friction test is shown. This sample was made up of four layers of E-type glass mat with 10 wt.% of quartz powder. Horizontal brighter strips correspond with the layers of the glass fiber reinforcement, while the vertical ones are the traces left after the friction belt.

The design of TT-4 tester also contributed to the decreased vibrations and thermal stabilization, namely, the registered increase in temperature in the contact area did not exceed 15°C in all tests. The ranges and standard deviations and thus measurement uncertainties are substantially lower than that of typical pin-on-disc tribometer, providing repeatability percentages measured for three different materials EV% = 13.4% [19]. 

From the results obtained for each sample, average friction force *F_fsr_* (N) was calculated. In order to avoid gross errors in the initial and final stages, the two first and the two last measurement results were excluded from further processing. Next, the friction work *A_f_* was calculated of as follows:

*A_f_* = *F_fsr_* × *s_d_*, (1)

where *s_d_* is a sliding distance [m].

Durability was measured in terms of specific wear work ***e_f_*** (J/g) and obtained from the following equation:

*e_f_* = *A_f_*/Δ*m*, (2)

where Δ*m* is the mass lost by the sample during the tribological test (g).

In order to detect outliers, the Dixon’s *Q* test was performed since it is particularly appropriate for small data sets [20]. The results were ranked in ascending order from *x*_1_ up to *x*_n_ and the respective lowest Q_min_ and highest Q_max_ observations were determined as follows:

*Q_max_* = (*x_n_* − *x_n−_*_1_)/(*x_n_* − *x*_1_), (3)

*Q_min_* = (*x_2_* − *x*_1_)/(*x_n_* − *x*_1_), (4)

where Δ*m* is the mass lost by the sample during the tribological test (g).

The largest values of *Q* were compared with the critical one, *Q_kr_,* dependent on the number of measurements and level of confidence [20]. In our experiments, level of confidence was assumed at 95%.

Among the methods of evaluating filled materials, the tensile strength testing is the most popular one [21]. However, considering the application of the tested material as a floor panels, it was decided that the tensile strength is not as important as the wear resistance. Since the bending strength seemed to be more important, it was measured according the DIN EN ISO 14125 standard and considered in the discussion. Moreover, hardness, shear strength, and impact strength were measured according to the respective standards EN ISO 6507-1:1997, PN-EN 2377:1994, and PN-EN ISO 179-1:2010. 

## 3. Results

Table 1 and Table 2 contain the results of the specific wear work ***e_f_*** (J/g) measured for the samples with two and four layers of glass mats, respectively. In general, the number of glass fiber layers provided no significant differences, but the dispersion of the results was higher for the four layer samples. In the Table 2, two outliers (bold) were identified and omitted in calculations of the average and standard deviation.

Statistical interpretation of these results is quite difficult since from six samples it was not possible unequivocally decide if the distribution is normal. Thus, Levene’s test was applied to assess the equality of variances for the obtained sets of variables. The performed test provided the ground to reject the null hypothesis that the population variances were equal. In this context, a non-parametric Kruskal–Wallis test was applied, which does not assume a normal distribution. For the significance level 0.1, the test revealed significant differences between the specimens with 3% and 6% of quartz powder in the four layer glass fiber reinforced composite (Table 2).

**Figure 4:**
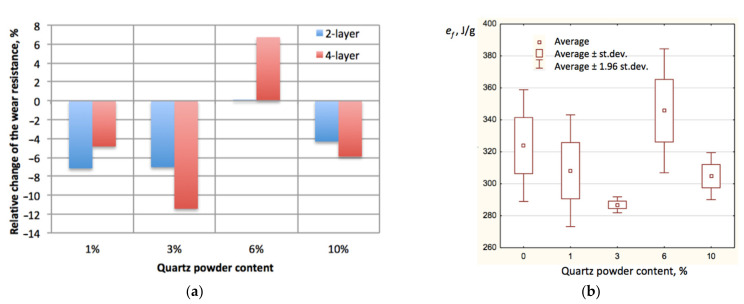
Specific wear work analysis: (**a**) Changes related to the samples with no quartz additions; (**b**) the average values with statistical error bars.

In terms of improvement, it can be concluded that smaller amounts of the quartz powder did not improve wear performance of the tested composite, as well as the increase in its content up to 10%. Figure 4a provides the graph of specific wear work changes related to the samples with no quartz additions and Figure 4b shows statistical errors of the obtained values.

Notably, only slight improvement took place after 6% of quartz powder addition to the two layer samples. Considering the dispersion of the results between the samples, the almost 10%, as shown in Table 1, improvement of 0.1% is negligible. Thus, it can be stated that addition of the quartz powder to the MC-DUR 1200VK reinforced with two layers of E-type glass mat does not improve its wear resistance.

In the case of the same material reinforced with four layers of glass mat, quartz powder caused favorable increase in the specific wear work only in the case of a 6% proportion. Either smaller or larger amounts caused decreases in the wear resistance of the composite.

**Figure 5:**
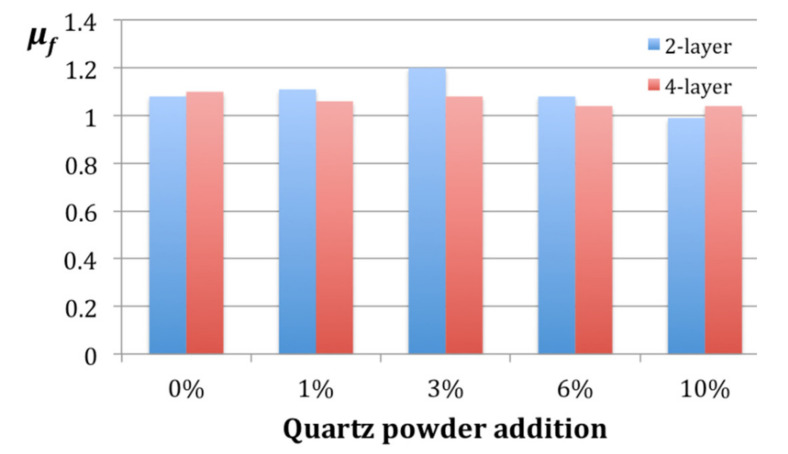
Friction coefficient *μ_f_* dependent on the quartz powder content.

However, the friction coefficient *μ_f_* does not exhibit such a dependence on the quartz powder percentage. Figure 5 provides the respective diagram.

For the two layer samples, the highest friction coefficient *μ_f_* = 1.2 was obtained for the 3% quartz powder content while the lowest *μ_f_* = 0.99 for 10% powder, with a difference above 17% of the maximum value. The four layer composite was much less sensitive to the powder content, since the differences between the largest and smallest *μ_f_* values were close to 5% and did not exhibit a clear trend.

**Figure 6:**
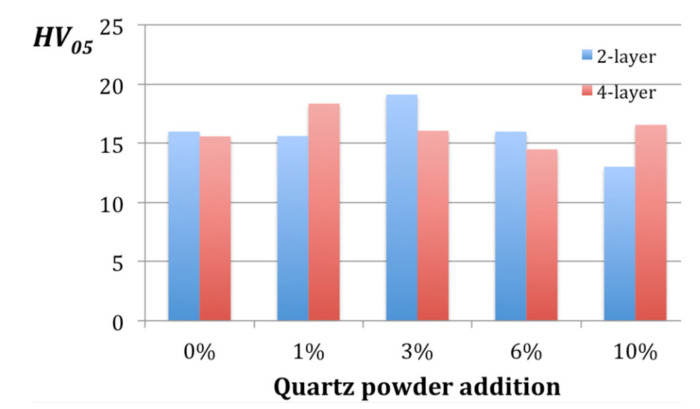
Microhardness *HV_05_* dependent on the quartz powder content.

Similarly, it is difficult to derive any strong conclusion about microhardness. Figure 6 presents the diagrams for *HV_05_* dependence on the powder content, but the maximum values for two layer and four layer samples do not correspond with the same powder proportion.

It can be observed that the composite with the two layer glass mat had the highest microhardness of 19.13 when it contained 3% of quartz powder, while the four layer composite exhibited quite a similar value of 18.37 with 1% of powder content. In contrast, the addition of 10% of powder caused an increase in hardness for four layer samples, but a decrease for two layer ones.

## 4. Discussion

The worsened properties of the composite with 10% of quartz powder can be attributed to the phenomena pointed out in the report [22]. The authors kept the percentage of silica filler below 7.5% to avoid weakening of the interfacial adhesion between the laminae that makes the composite more fragile. However, other results do not confirm their suggestions that the SiO_2_ filler addition unequivocally improves hardness, impact strength, and wear resistance.

**Figure 7:**
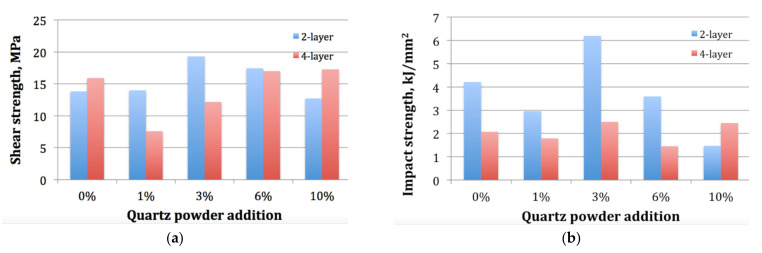
Strength of the tested 2-layer and 4-layer samples with different proportions of the quartz powder: (**a**) shear strength; (**b**) impact strength.

From the perspective of the application of the tested polymer composite as a floor panel, the wear resistance is not the only parameter that must be considered. It is widely recognized that the composite laminates are prone to the delamination phenomenon [23]. Thus, to prevent the floor panel from damages, its material should reflect high shear and impact strength. However, neither shear strength nor impact strength reflected a clear improvement at 6% addition of the quartz powder, which took place in case of wear resistance. Figure 7 shows the graphs of the strength for the tested samples with respect to the quartz powder content.

From the Figure 7, it can be observed that the composite of best wear resistance, namely, four layer with 6% of quartz powder, did not exhibit the highest strength. Its shear strength (Figure 7a) was lower than that of the two layer samples with similar powder proportion and with 3% of powder. Among four layer samples, very close results were obtained for the samples with 10% of powder and insignificantly lower strength was reflected in the samples with no powder added. The impact strength of this composite was the lowest among all samples and similar to that of the two layer with 10% of powder. In comparison to the four layer samples with no powder added, additions of 6% of quartz powder increased shear strength of the composite by 7%, but worsened its impact strength by 30%.

The results on interlaminar shear strength are somehow related to the other studies indicating its increase for 6% content of SiO_2_ in the E-glass reinforced epoxy composite [24]. It should be noted, however, that in [24] the shear strength increased from 9.8 MPa without SiO_2_ up to 11 MPa of 6 wt.% while our composites exhibited a respective increase from ca. 15 MPa up to ca. 17 MPa.

Moreover, our study revealed that the pattern of the quartz powder effect is different in case of two layer and four layer tested composites. For the two layer composite, the highest shear strength was obtained at a 3% proportion of the quartz powder, which was not the case for the four layer samples. In fact, both shear and impact strength were close to its highest value for 10% of quartz addition to the four layer composite, but similar shear strength were reflected in the 0% and 6% samples and similar impact strength was reflected in the 3% sample. Therefore, since the main criterion in our study was the specific wear work, the four layer composition with 6% powder can be considered the best material obtained in the experiments despite reduced hardness and impact strength. Additionally, this conclusion is supported by the bending strength measurement, which provided highly dispersed results from 66 MPa up to 126 MPa with no clear dependence on the quartz proportion, but its highest value was obtained for the four layer composition with 6% of quartz filler.

The mechanical and physical performances of bulk polymer laminates are affected greatly by the interfacial behavior and interaction between glass fiber and matrix [25]. It can be assumed that the processes of matrix destruction during the polymer composite damage are dependent on the phenomena that take place between the phases of a composite and often on the loss of adhesion between the elastic resin and stiff hard reinforcement [26]. From this perspective, it can be explained why the polymer composite with four layer reinforcement exhibits lower impact strengths than the two layer one. Other studies [27] reported an almost proportional decrease in the impact strength with increase in silica content. In their case, reduction was by 38% for 10% SiO_2_ content, while in our research, two layer composites exhibited much larger respective differences, but the four layer one reflected a slight increase in impact strength.

## 5. Conclusions

The results of the research on silica addition to the glass fiber reinforced polymer composite indicated a much more complicated effect of the filler on the composite properties than was expected. No clear trend can be derived for the effect of SiO_2_ percentage on the composite performance and statistical analysis did not reveal any unequivocal dependence. 

In the study, the main parameter considered was the wear resistance due to the destined application of the tested material for floor panels. The wear was assessed using the specific wear work determined by the novel tribotester with friction band. This method provided better repeatability than a typical pin-on-disc test due to the reduced vibration and temperature effect, removal of debris, and avoidance of the repeated contact of the worn surface with the tested specimen. From the wear tests, it can be concluded that only the composite with four layers of glass fibers and 6 wt.% of the quartz powder exhibited an improvement in wear resistance, reaching the highest specific wear work of *e_f_* = 345 J/g. The bending strength of the composite with this filler proportion, namely 126 MPa, was the highest among all the tested specimens. Moreover, other specimens had worsened characteristics than the ones without silica additions. Statistically, no significant differences were found between the results obtained for respective proportions of quartz powder with two and four layers of glass reinforcement. Thus, it can be stated that the four layer tested composite provided no considerable improvement compared to the two layered one.

On the other hand, the highest friction coefficient’s, which is microhardness *HV_05_*, shear strength and impact strength were attained for the composite with two layers of glass fibers and 3 wt.% of the quartz powder. The results indicate the need of individual purpose-dependent decision making in the design of the glass fiber reinforced composites with quartz powder filler.

## Figures and Tables

**Table 1:** Specific wear work ***e_f_*** (J/g) obtained for the samples with two layers of E-type glass reinforcement.

Sample No.	Powder Percentage				
	0%	1%	3%	6%	10%
1	308.82	289.46	343.33	299.05	327.17
2	329.35	292.06	306.86	315.34	295.59
3	335.02	305.05	285.62	303.83	296.26
4	281.43	315.13	259.84	291.32	274.50
5	326.90	262.13	320.33	375.53	344.12
6	332.36	312.81	263.43	330.46	293.52
Average	318.98	296.11	296.57	319.25	305.19
Standard deviation	20.59	19.68	32.94	30.77	25.49
Relative standard deviation	6.45%	6.64%	11.11%	9.64%	8.35%

**Table 2:** Specific wear work ***e_f_*** (J/g) obtained for the samples with four layers of E-type glass reinforcement.

Sample No.	Powder Percentage				
	0%	1%	3%	6%	10%
1	293.74	288.76	288.52	420.98	333.31
2	279.06	280.38	296.11	324.56	293.75
3	396.35	342.25	**344.05**	329.08	306.61
4	354.75	381.42	283.44	291.65	295.19
5	311.97	284.17	282.52	388.05	**397.96**
6	307.55	272.18	283.60	319.94	294.96
Average	323.90	308.19	286.84	345.71	304.76
Standard deviation	43.66	43.70	5.69	48.52	16.79
Relative standard deviation	13.48%	14.18%	1.98%	14.03%	5.51%

## Data Availability

Data are available upon request due to privacy restrictions.
